# Topological features of spike trains in recurrent spiking neural networks that are trained to generate spatiotemporal patterns

**DOI:** 10.3389/fncom.2024.1363514

**Published:** 2024-02-23

**Authors:** Oleg Maslennikov, Matjaž Perc, Vladimir Nekorkin

**Affiliations:** ^1^Federal Research Center A.V. Gaponov-Grekhov Institute of Applied Physics of the Russian Academy of Sciences, Nizhny Novgorod, Russia; ^2^Faculty of Natural Sciences and Mathematics, University of Maribor, Maribor, Slovenia; ^3^Department of Medical Research, China Medical University Hospital, China Medical University, Taichung City, Taiwan; ^4^Complexity Science Hub Vienna, Vienna, Austria; ^5^Department of Physics, Kyung Hee University, Seoul, Republic of Korea

**Keywords:** spiking neural network, target spatiotemporal pattern, supervised learning, reservoir computing, spike metrics, persistent homology

## Abstract

In this study, we focus on training recurrent spiking neural networks to generate spatiotemporal patterns in the form of closed two-dimensional trajectories. Spike trains in the trained networks are examined in terms of their dissimilarity using the Victor–Purpura distance. We apply algebraic topology methods to the matrices obtained by rank-ordering the entries of the distance matrices, specifically calculating the persistence barcodes and Betti curves. By comparing the features of different types of output patterns, we uncover the complex relations between low-dimensional target signals and the underlying multidimensional spike trains.

## 1 Introduction

The challenge of understanding how spatiotemporal patterns of neural activity give rise to various sensory, cognitive, and motor phenomena in nervous systems is a significant task in computational and cognitive neuroscience. A prominent paradigm for proposing hypotheses about potential mechanisms involves training recurrent neural networks on target functions, considering biological constraints and relating dynamic and structural features in the obtained networks to characteristics of inputs and outputs (Sussillo, 2014; Barak, 2017; Yang and Wang, 2020; Amunts et al., 2022; Maslennikov et al., 2022). This approach is in line with a more traditional research domain of finding dynamical mechanisms underlying various spatiotemporal patterns observed in the brain (traveling waves, oscillatory rhythms in different frequency domains, chaotic or disordered spike firing etc.) (Liu et al., 2022a,b; Yu et al., 2023a,b), which is highly interdisciplinary and borrows different approaches from physics and mathematics.

An interdisciplinary approach has emerged at the intersection of computational neuroscience, machine learning, and non-linear dynamics. This approach considers similarities in time-dependent processes in biological brains and artificial neural networks as consequences of computations through population dynamics (Marblestone et al., 2016; Hassabis et al., 2017; Cichy and Kaiser, 2019; Vyas et al., 2020; Dubreuil et al., 2022; Ramezanian-Panahi et al., 2022). Works in this direction focus on training networks of rate neurons on cognitive-like and sensorimotor neuroscience-based tasks, revealing computational principles for completing target tasks in terms of dynamics, functional specialization of individual neurons, and coupling structure (Sussillo and Abbott, 2009; Mante et al., 2013; Sussillo and Barak, 2013; Abbott et al., 2016; Chaisangmongkon et al., 2017; Maslennikov and Nekorkin, 2019, 2020; Maslennikov, 2021).

Real neural networks differ from rate-based models, primarily in that they produce sequences of action potentials or spikes. To account for this important aspect, another class of neural networks—spiking ones—has been developed. On the one hand, they are more biologically realistic in producing firing patterns of a similar structure, leading to a more thorough comparison between artificial and biological spiking networks in their dynamics and structural mechanisms of functioning (Eliasmith et al., 2012; Gilra and Gerstner, 2017; Kim et al., 2019; Lobo et al., 2020; Pugavko et al., 2020, 2023; Amunts et al., 2022). On the other hand, spiking networks are the next-generation class of neural networks that are capable of energy-efficient computations when performed on specialized neuromorphic chips (Schuman et al., 2022). Although they can be obtained from convenient neural networks using some conversion techniques, to take their full advantage, one needs to use specific algorithms to train them (Demin and Nekhaev, 2018; Neftci et al., 2019; Tavanaei et al., 2019; Bellec et al., 2020; Dora and Kasabov, 2021). Spiking neural networks have demonstrated their capabilities in various applications including processing signals of different modalities (Bing et al., 2018; Auge et al., 2021; Yamazaki et al., 2022), robotics (Lobov et al., 2020, 2021; Angelidis et al., 2021), and more generally in brain-inspired artificial intelligence tasks and brain dynamics simulations (Zeng et al., 2023).

As in the case of biological neural systems, artificial spiking networks are hardly interpreted when they perform complex motor or cognitive-like tasks. While rate-based neural networks organize their dynamics along smooth manifolds which can be often studied as projections to low-dimensional subspaces, for spiking networks, such procedure in general may not be done (Muratore et al., 2021; Cimeša et al., 2023; DePasquale et al., 2023). One of the promising approaches to characterize spiking patterns is methods of algebraic topology. Such tools as persistent homology analysis have been used in relating spike patterns with functions of neural networks (Dabaghian et al., 2012; Petri et al., 2014; Curto, 2017; Bardin et al., 2019; Santos et al., 2019; Sizemore et al., 2019; Naitzat et al., 2020; Billings et al., 2021; Guidolin et al., 2022)—both biological and artificial—and more widely for studying topological aspects of dynamical systems (Maletić et al., 2016; Stolz et al., 2017; Salnikov et al., 2018; Myers et al., 2019).

In this study, we explore topological features in spiking neural networks trained to generate low-dimensional target patterns. We study recurrent networks in the class of reservoir computers (Maass et al., 2002; Lukoševičius and Jaeger, 2009; Sussillo, 2014) where training only occurs at the output connections. After training, the networks produce spiking dynamics which underlie the generation of output patterns, and our goal is to study how topological features of the spike trains carry information about output patterns in terms of persistence barcodes and Betti curves. In Section 2, we present the system under study and the mkey findings of our study. Section 3 sums up the results and Section 4 gives particular details of the model and methods.

## 2 Results

### 2.1 Training recurrent spiking neural networks to generate target outputs

**Figure 1:**
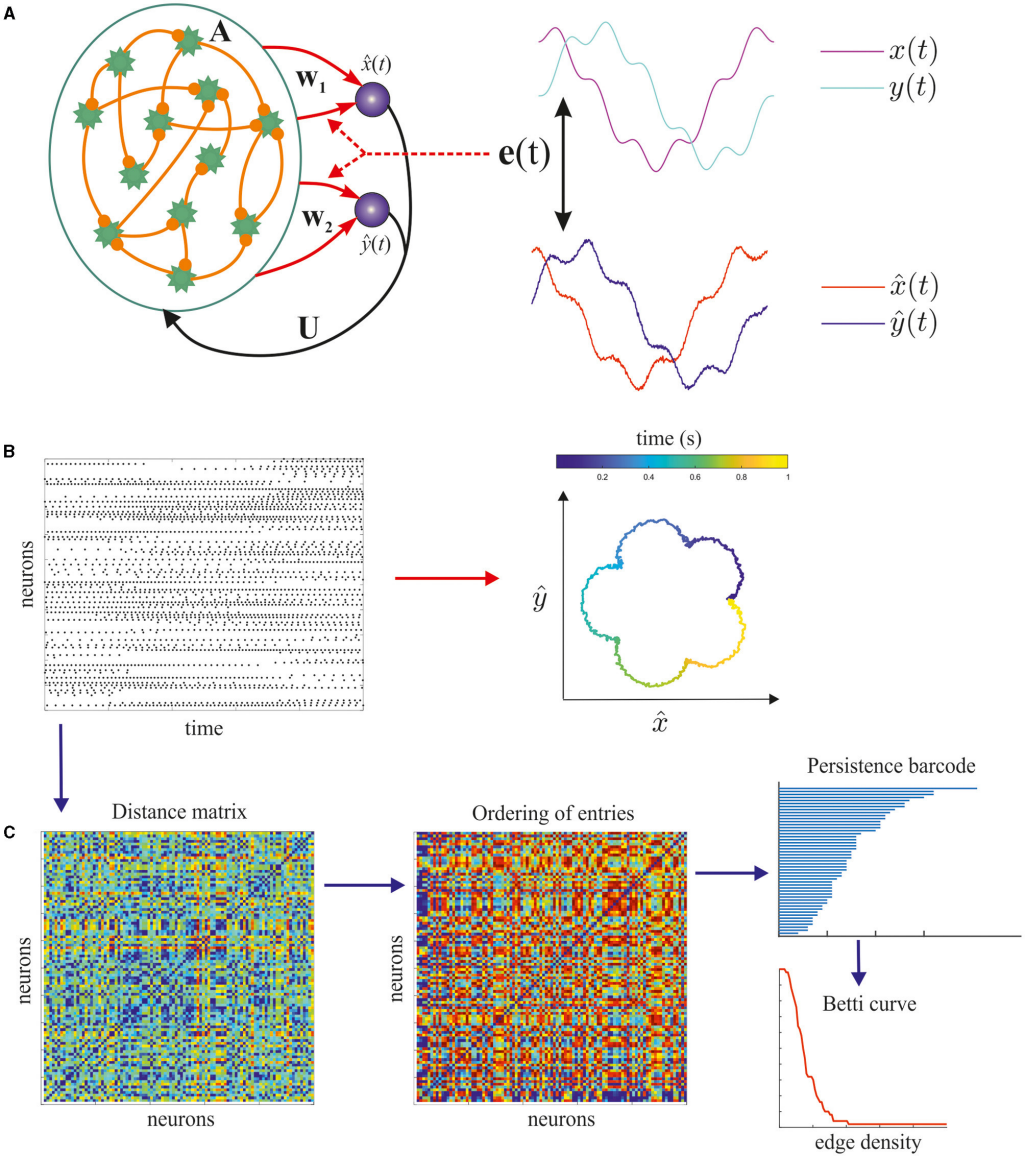
Flowchart of the study. **(A)** Training a spiking neural network to generate a target trajectory at the output by the FORCE method. **(B)** After training, the self-sustained spiking patterns support the generation of the pattern of interest. **(C)** Spike trains are analyzed in several steps. First, matrix **D** of the Victor–Purpura distances is calculated. Second, matrix **M** obtained by rank ordering the entries of **D**. Finally, we apply several approaches of the algebraic topology, namely, we compute the persistent homology of the rank-ordered matrix obtaining persistence barcodes and Betti curves which give topological signatures of the spiking patterns.

We consider recurrent networks of spiking neurons trained to generate two-dimensional spatiotemporal signals and study how topological signatures of their spike patterns relate to the readout activity. The pipeline of our study is schematically presented in Figure 1. The neurons are randomly connected with sparse links whose weights are drawn from Gaussian distribution and kept fixed. The structure of links is determined by the adjacency matrix **A**. Two scalar outputs (which can be considered as one vector output) $\hat{x}\left(t\right)$ and ŷ(*t*) linearly read out the filtered spiking activity of the recurrent network via output weight vectors **w**_1_ and **w**_2_. The output signals also send feedback connections given in matrix **U** to the recurrent neural network. While the feedback links are initialized and kept fixed as the recurrent ones, the output links are changed during training in order to minimize the error **e**(*t*) between the target pattern [*x*(*t*), *y*(*t*)] and the real output signals $\hat{x}\left(t\right)$, ŷ(*t*), see Figure 1A. Such training setting is a particular case of the reservoir computing paradigm in which the weights only in the last layer are trained. In this study, training is made by the FORCE method (see details in Section 4).

The networks we study consist of leaky integrate-and-fire neurons with an absolute refractory period, and the output trajectories are chosen as closed polar curves, see details in Section 4. After training, the networks are capable of producing these two-dimensional signals which can be treated as target motor patterns produced by spiking activity, see Figure 1B. Our purpose is to relate the spiking patterns of the trained neural networks with the target trajectories. The output signals are produced as weighted sums of the firing-rate activity, but the question is to what extent the detailed spike trains—not the averaged rates—are responsible for producing target patterns? To answer this question, we measure how dissimilar individual spike trains are from each other. There are many correlation-based characteristics which enable to quantify similarity between signals produced by neurons, but they do not capture the fine structure of spike timing. Here, we adopt the method proposed by Victor and Purpura to compute a special quantity—the Victor–Purpura (VP) distance which considers a spike sequence as a point in some metric space. We calculate a matrix of VP distances where each entry (*i, j*) quantify how dissimilar or distant are the spike trains produced by the *i*-th and *j*-th neurons. After that we transform the obtained distance matrices by rank ordering their entries. Then, we apply the method of algebraic topology—the persistent homology—to the latter matrix and obtain the so-called persistence barcodes and Betti curves, see Figure 1C. These topological signatures are detailed characteristics of spike patterns responsible for the generation of the patterns under study, so we study how the spiking topology features relate to low-dimensional output signals.

**Figure 2:**
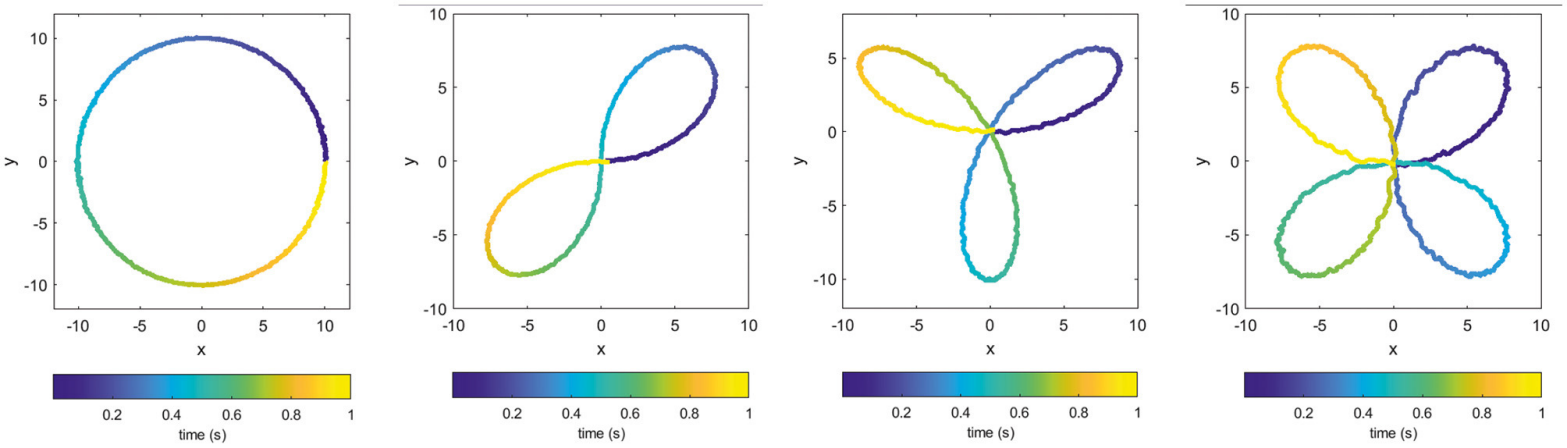
Examples of output trajectories [*x*(*t*), *y*(*t*)] produced by the trained recurrent spiking neural network: (from left to right) a circle, two-petal, three-petal, and four-petal polar roses.

We applied the proposed pipeline to different types of teacher signals but in order to conveniently visualize and easily understand topological features of the target patterns themselves, we show the results for four polar closed curves having different number of holes, as shown in Figure 2. The figures in fact show the real outputs perfectly matching the target signals and having a small noisy component resulting from the spiking nature of the network.

**Figure 3:**
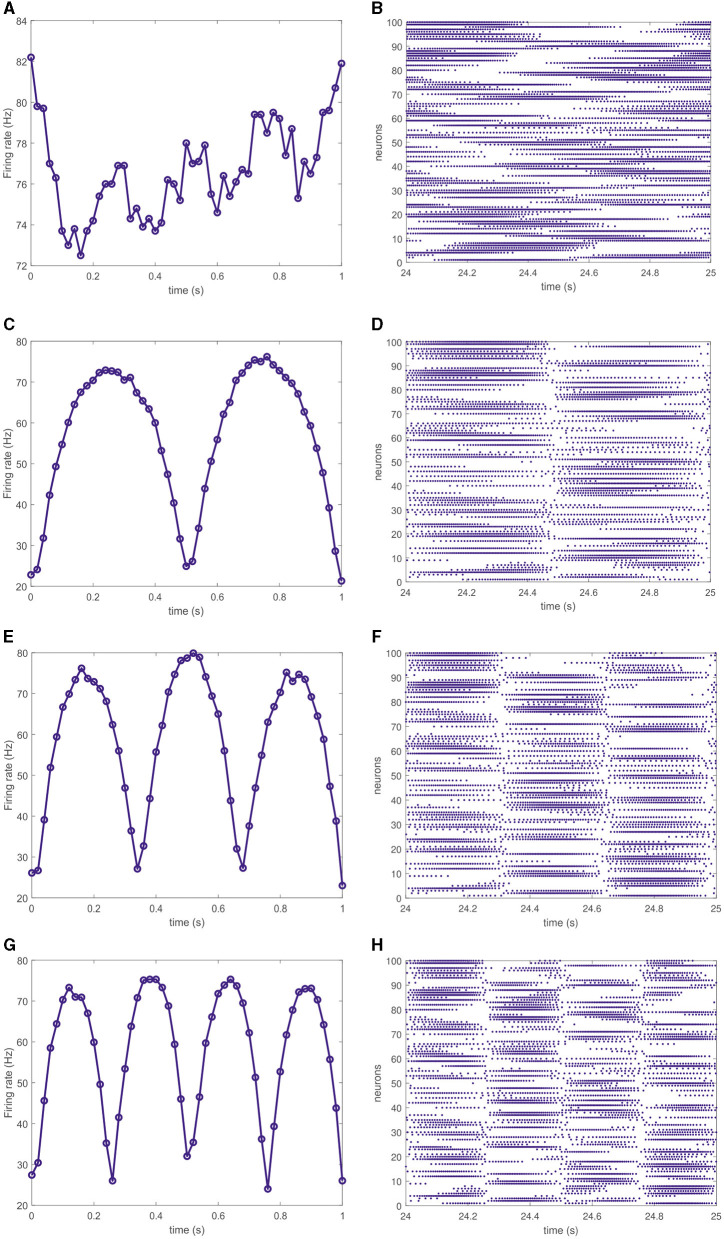
Instant firing rate of the full network averaged over 20 ms for four different target outputs shown in Figure 2 (left column). Corresponding spike trains of 100 randomly chosen neurons (right column). For the circle target output, the firing rate changes within the interval of 72–84 Hz **(A)** and the corresponding spike train does not exhibit any distinct phases **(B)**. For the target patterns in the form of polar roses **(C, E, G)**, the network firing rate varies within the band of 20–80 Hz making discernible rise-fall excursions for each petal of the output trajectory. The corresponding spike trains **(D, F, H)** contain the same number of distinct phases as the number of petals in the target pattern.

In terms of individual spiking activity, different neurons in the trained networks fire at various rates. Namely, within particular segments of the target pattern some neurons actively generate action potentials while other are silent and start to fire in further segments of the pattern. The overall network activity can be characterized by the mean firing rate as the average number of spikes per second and per neuron. Figures 3A, C, E, G shows evolving mean firing rates in the networks, producing four corresponding target patterns, as shown in Figure 2. Notably, for all the cases, the firing rate changes within the interval of 20–80 Hz except the simplest circle target pattern where the firing rate varies within the narrow interval of 72–84 Hz. For target signals in the form of multipetal roses, the firing rate increases and decreases following each petal, see Figures 3C, E, G. The corresponding spike rasterograms shown in Figures 3B, D, F, H indicate that rises and falls of the mean firing rate are supported by the activity of different neurons. Therefore, although output patterns are produced by filtered spikes, i.e., instant firing rates of neurons, one cannot relate the rate activity with the properties of the output pattern in a direct way. Moreover, the temporal structure—not only rates—of spikes is responsible for generating output patterns of different forms.

### 2.2 Distance matrices for spike trains in the trained neural network

To compare different neurons in terms of dissimilarities between their spike trains, we apply the method proposed by Victor and Purpura (see Guidolin et al., 2022 and works cited therein). Namely, this method endows a pair of spike trains with a notion of distance. This is in contrast with the frequently used method to quantify pairs of neuronal responses by the rate-based correlations. Spike trains of some finite length are considered as points in an abstract space where a special metric rule is defined which assigns a non-negative number *D*_*ij*_ to each pair of points *i*, *j*. The Victor–Purpura (VP) distance has several basic properties required to be a true metric, namely, it vanishes only for the pair of identical spike trains (*D*_*ii*_ = 0) and it is positive otherwise (*D*_*ij*_ > 0, *i* ≠ *j*), it is symmetrical (*D*_*ij*_ = *D*_*ji*_), and it fulfills the triangle inequality (*D*_*ik*_ ≤ *D*_*ij*_ + *D*_*jk*_). The VP distance between spike trains is defined as the minimum cost of transforming one spike train into the other via the addition or deletion of spikes, shift of spike times, or change in the neuron of origin of the spikes. Each modifying move is characterized by cost *q* which controls the timescale for shifts of spikes. In general, there is a family of distances defined in this way which can capture the sensitivity to the neuron of origin of each spike. Here, we use the basic VP metric which assigns cost *q* = 1 per unit time to move a spike (see details in Section 4).

**Figure 4:**
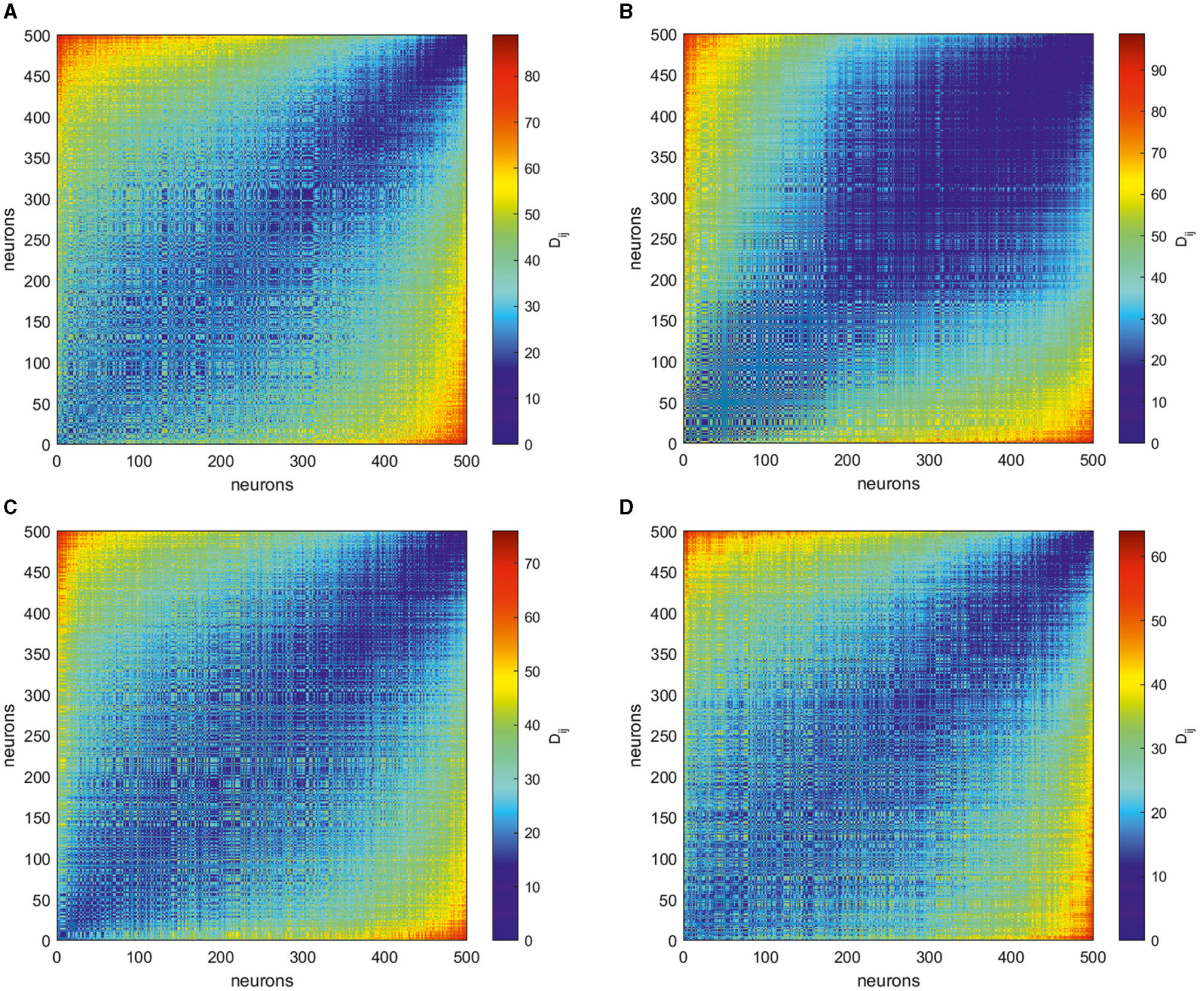
Matrices of the Victor–Purpura distance **D** = [*D*_*ij*_] obtained for spike trains underlying the generation of four different target outputs in Figure 2: **(A)** a circle, **(B)** two-petal, **(C)** three-petal, and **(D)** four-petal polar roses. More distant neurons shown by red entries fire most dissimilar spike trains while less distant ones given by blue entries generate comparable spike patterns. The matrices show that different target patterns require special organization of spike trains.

For each of the four target patterns illustrated here, we collect spike trains $S^{\left(i\right)}=\left[t_{1}^{\left(i\right)},t_{2}^{\left(i\right)},\ldots ,t_{s_{i}}^{\left(i\right)}\right]$, *i* = 1, …, *N* for the period of 1 s (the duration of the target generation). Then, we calculate the VP distances for each pair of spike trains and obtain matrices **D** = [*D*_*ij*_], as shown in Figure 4. These matrices are symmetric and reflect the intricate temporal structure of the spike patterns supporting corresponding output trajectories. Notably, even at this stage, one can make several qualitative conclusions about differences in spike patterns relating to different target outputs. Despite similar ranges of the firing rate varying for all targets, as shown in Figures 3A, C, E, G, matrices of VP distances for their spike trains have distinct differences. The simplest circle target correspond to the matrix where most of entries take similar values in the middle of the range of possible distances (see Figure 4A). For multi-petal closed trajectories, the maximum distance becomes smaller for increasing the number of holes in the output patterns, cf. Figures 4B–D. Moreover, less-petal polar roses require more neurons that produce less distant spike trains.

**Figure 5:**
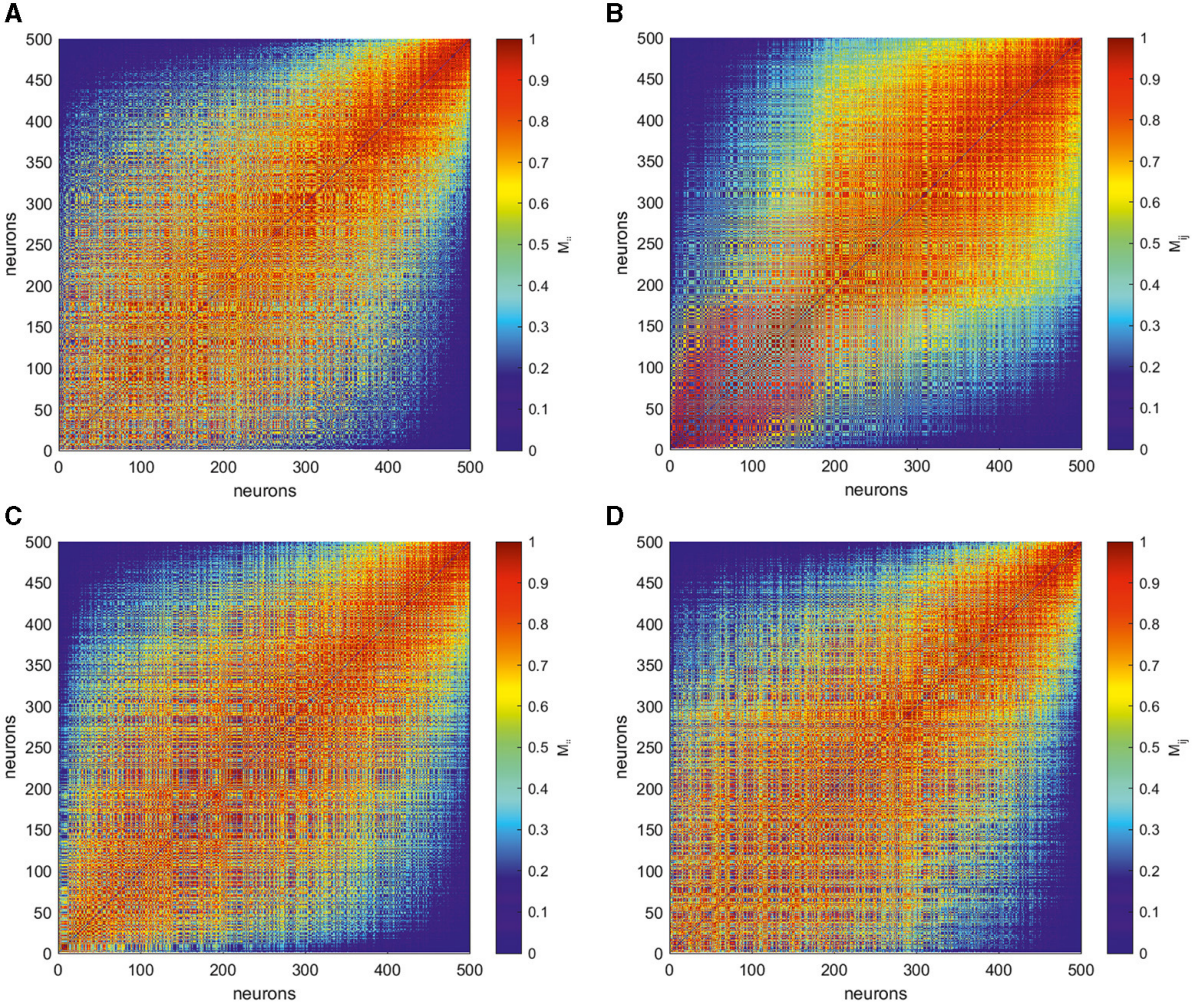
Matrices **M** = [*M*_*ij*_] produced by rank ordering and normalization of the entries in the corresponding VP matrices **D** = [*D*_*ij*_] shown in Figure 4 for four different target outputs, as shown in Figure 4: **(A)** a circle, **(B)** two-petal, **(C)** three-petal, and **(D)** four-petal polar roses. The smaller entries (blue) of these matrices **M** correspond to most dissimilar spike trains while the larger ones (red) indicate the closest neurons in term of VP distance. The neurons here are reordered based on the individual average firing rates, thus the units with a smaller index produce more spikes during trials than those with larger indices. The form of the matrices emphasize that neurons that produce spikes with close firing rates are closer to each other than to those producing a greatly different number of spikes. However, different target patterns are characterized by individual signatures.

To get more insight into intricate structure of spike trains, following Giusti et al. (2015) and Guidolin et al. (2022), we transform the obtained matrices by rank ordering their entries. Namely, given a matrix of VP distances *D*_*ij*_ with zeros on its main diagonal, we consider the entries of its above-diagonal part and replace them by natural numbers 0, 1, …  in ascending order of their value. The below-diagonal part of the rank-ordered matrix is completed symmetrically, thus resulting in the rank-ordered matrix **M** = [*M*_*ij*_]. Thus, the more VP distance *D*_*ij*_, the smaller the corresponding entry *M*_*ij*_. Finally, we normalize the entries of the latter matrix by the maximum *N*(*N* − 1)/2 and reindex in descending order of the individual firing rate of the corresponding neurons, thus obtaining matrix **M** = [*M*_*ij*_] for four target patterns of interest, as shown in Figure 5.

The smallest values of *M*_*ij*_ correspond to the pairs of spike trains which are the most dissimilar, and the highest entries indicate most closest neurons in the Victor–Purpura sense. Notably, less active neurons with largest indices are the most similar between each other, see the up-right parts of matrices in Figure 5, and far away from the most active neurons. The down-left part of rank-ordered matrices correspond to neurons which fire most actively during the task implementation and thus mostly contributing to the output patterns. Their spike trains show highly complicated structure which depends on the target pattern. To characterize the structure of the relations between the core neurons, we take 100 most active ones and study the topological features of the graph of their rank-ordered VP distances.

### 2.3 Persistent homology of rank-ordered matrix

Most frequently used tool in topology data analysis is persistent homology. While initially this framework has been developed for static data sets, many ideas are adopted to studying time-varying dynamic data (Petri et al., 2014; Curto, 2017; Stolz et al., 2017; Myers et al., 2019; Santos et al., 2019). Homology refers to certain topological properties of data, whereas persistence reflects the properties which are maintained through multiple scales of the data.

**Figure 6:**
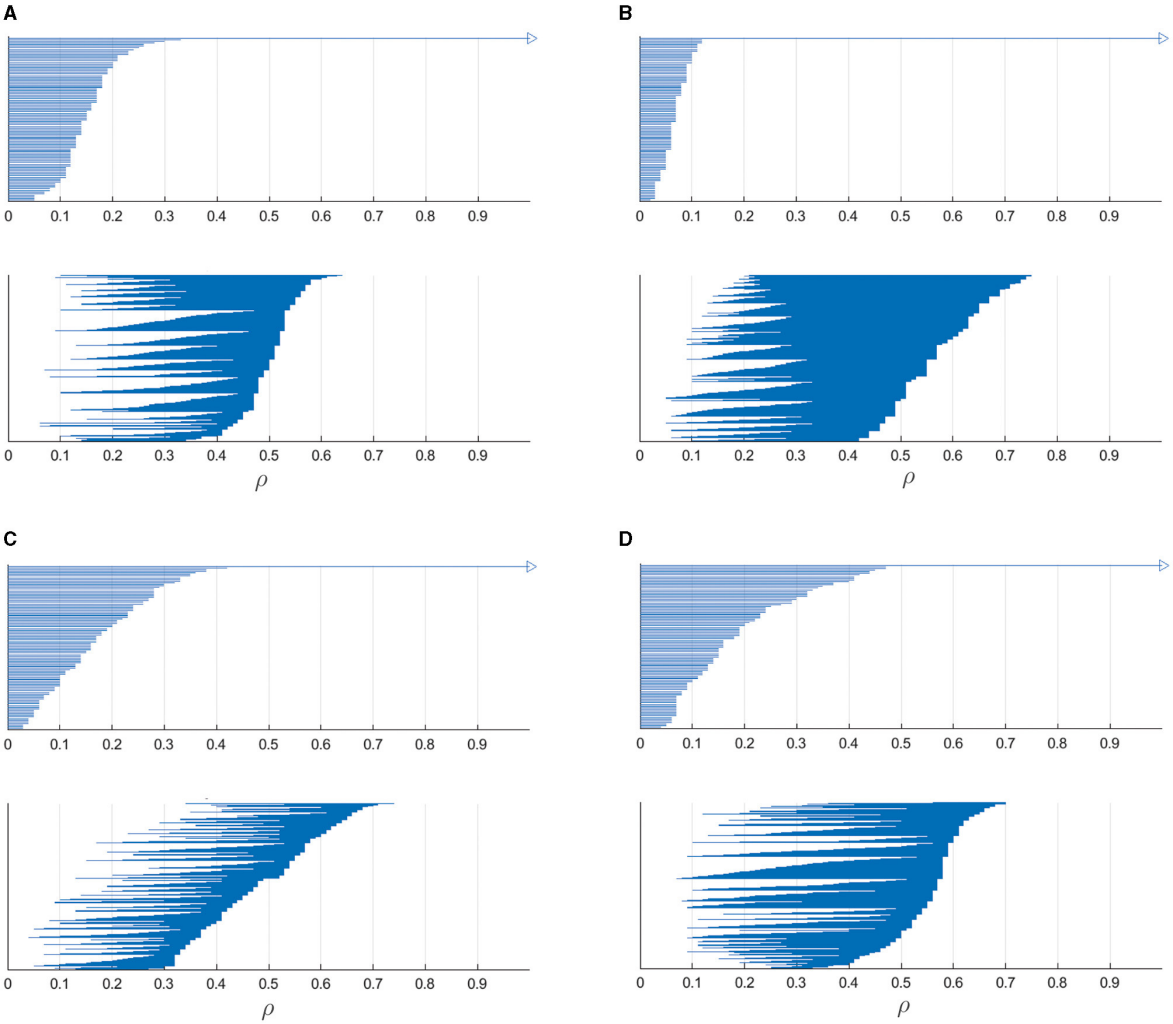
Barcodes showing the persistence of zero-dimensional and one-dimensional simplices for 100 most active neurons taken from the matrices shown in Figure 5 for four different target outputs: **(A)** a circle, **(B)** two-petal, **(C)** three-petal, and **(D)** four-petal polar roses. Bars in the top subfigures show existence of zero-dimensional simplices and those in bottom subfigures indicate the birth and death of one-dimensional simplices. These persistence barcodes reflect complex topological structure of spike trains produced by the neural networks performing particular output trajectories.

Our set of neurons and the corresponding spike trains form a point cloud of vertices for which a notion of distance collected in **M** is determined. This set of vertices form zero-dimensional simplices while one-dimensional simplices are the edges between them. Imagine each vertex is surrounded by a circle of radius ρ, and this value is gradually increasing starting from zero. If the circle centered in vertex *i* has radius ρ larger than the distance *M*_*ij*_ to vertex *j*, the pair of nodes *i* and *j* are considered coupled and form a one-dimensional simplex. Initially (ρ = 0), all the vertices are isolated, hence they form a set of zero-dimensional simplices and there is no one-dimensional simplices. When radius becomes such that some pair of vertices become coupled, a new one-dimensional simplex appears while zero-dimensional simplices corresponding to the vertices disappear. Such gradual increase of the radius is called filtration and can be presented in the form of persistence barcodes, as shown in Figure 6. Here, parameter ρ indicates the radius of circles surrounding each vertex and the bars show zero- and one-dimensional simplices: at which values of ρ they appear and when dissapear.

In the top subfigures, the number of initially existing zero-dimensional simplices is equal to the chosen number of most active neurons (100), and with increasing filtration parameter ρ, the number of bars gradually decreases, finally leading to one remaining simplex corresponding to the connected component which contains all the vertices. In the bottom subfigures, the barcodes show the birth and death of one-dimensional simplices with increasing filtration parameter. Altogether, these persistence barcodes describe topological signatures of spike trains relating to most active neurons which mostly contribute to generating particular target outputs.

**Figure 7:**
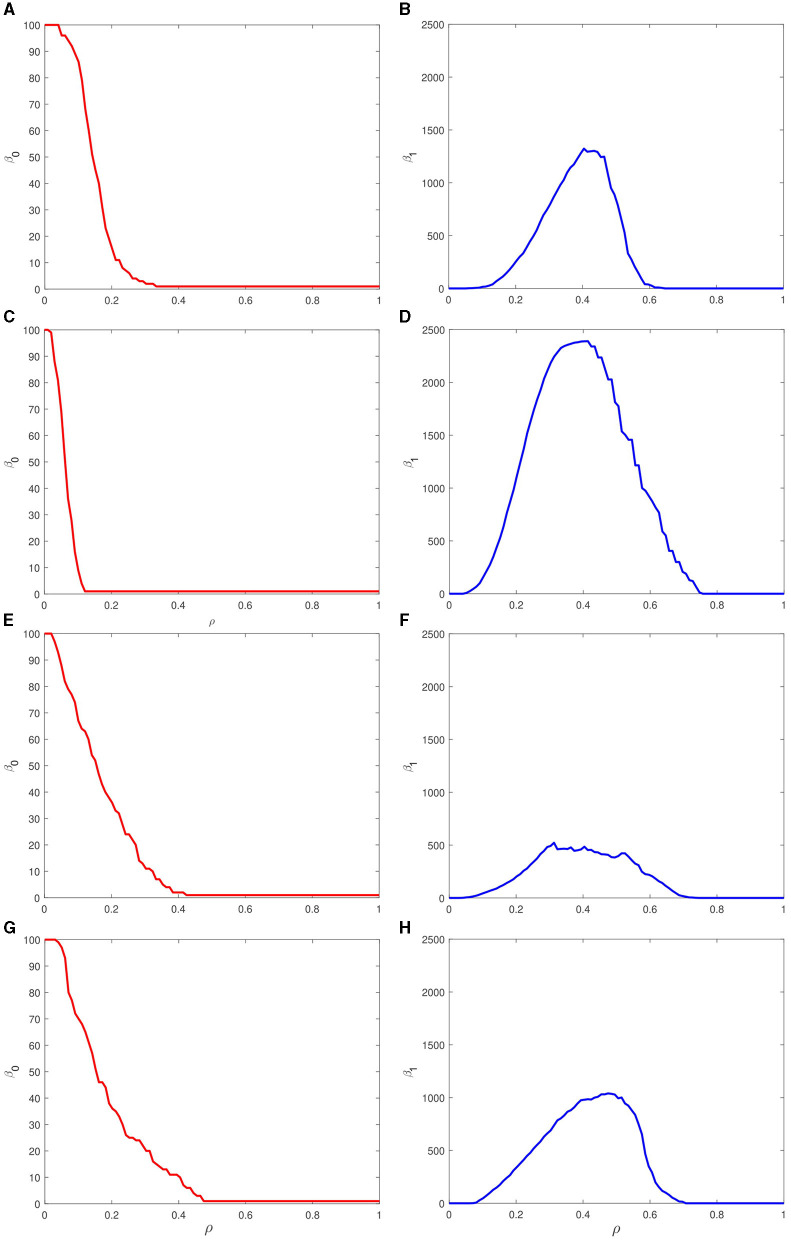
Betti curves for zero-dimensional (left column) and one-dimensional (right column) simplices associated with increasing filtration of the persistence barcodes shown in Figure 6 for four different target outputs: **(A, B)** a circle, **(C, D)** two-petal, **(E, F)** three-petal, and **(G, H)** four-petal polar roses. Each curve indicates the number of simplices of particular dimension with varying filtration parameter ρ.

To summarize the filtration process, the number of persisting topological invariants for particular values of ρ is plotted in the form of Betti curves shown in Figure 7. These generalizing curves show the course of emergence and disappearance of zero-dimensional (left column) and one-dimensional (right column) simplices in the point clouds formed by spike trains of most active neurons. The number of zero-dimensional simplices shows the distinct monotonically decreasing dependence on the filtration parameter. The most sharp drop is observed for the two-petal target pattern (Figure 7C) while the one-, three-, and four-hole trajectories result in a smoother decrease until the threshold value of ρ, in turn, slightly increases around ρ = 0.4 with increasing number of holes, cf. Figures 7A, E, G. The number of one-dimensional simplices shows a more intricate structure where the maximum is in complex dependence on the features of the target output. The largest one relates to the two-petal polar rose and the smallest maximum to the three-petal one while the remaining targets lead to the plots with with similar maxima.

Comparing these figures with the corresponding barcodes in Figure 6 and matrices in Figure 5, one concludes that the chosen target patterns which have easily explainable forms in terms of topology require spike trains which are characterized by topologically complex characteristics. We found that there is no direct correspondence between Betti numbers of generated trajectories and simplicial complexes built upon the spike trains. However, topological analysis according to the proposed pipeline allows us to extract valuable information about coding principles of spikes at the level of precise firing timing and the topological relations between spike trains of different neurons.

## 3 Discussion

We applied algebraic topology methods, specifically persistent homology, to characterize geometry of spike trains produced by recurrent neural networks trained to generate two-dimensional target trajectories. We considered several easily interpreted two-dimensional closed trajectories as the target patterns for training recurrent spiking networks. The FORCE method is used for supervised learning which is a particular framework of reservoir computing, where weight modification occurs at the output layer while recurrent connections are randomly initialized and kept fixed. In addition, the random feedback connections from the output provide indirect low-rank perturbation to the recurrent matrix, thus creating the modified effective coupling architecture capable of producing target patterns. The neural spike trains in the trained networks were considered as points in some metric space, where the distances between them were calculated as cost-based Victor–Purpura quantities. We rank-ordered the measured distances and chose one hundred most active neurons for which we performed persistent homology analysis. We plotted persistence barcodes and Betti curves, which characterize how specific topological objects in the spiking data were preserved under continuous transformation. We find complicated relation between topological characteristic of spike trains and those of target patterns. The novelty of our study is that we apply persistence homology methods to the spiking networks trained to autonomously generate planar output trajectories. Previously, such methods were mostly applied to the neural networks performing navigation tasks and consisting of neurons which fire preferentially in particular locations in the environment—place fields. Thus, one was able to find a one-to-one correspondence between topological features of the environment and those of spiking patterns. Our study is an attempt to establish regularities in a more general case where generated trajectories do not carry navigation information yet have clear topological interpretation.

## 4 Methods

### 4.1 Spiking neural network and target outputs

We consider a recurrent spiking neural network consisting of *N* leaky integrate-and-fire neurons whose activity is projected into *M* scalar outputs, see Figure 1. Autonomous dynamics of the spiking network is described by the following system (Nicola and Clopath, 2017):


(1) $$\begin{array}{l} \tau_{m}\frac{dv_{i}}{dt}=v_{rest}-v_{i}+I_{bias}+\overset{N}{\underset{j=1}{\sum }}a_{ij}r_{j}, \end{array}$$

where *v*_*i*_ is a membrane potential (voltage) of the *i*-th neuron, τ_*m*_ is a time constant for the voltage relaxation, *v*_*rest*_ is the resting voltage, *I*_*bias*_ is an input bias current (the default value in our numerical experiments is *I*_*bias*_ = 0), and *a*_*ij*_ are the weights describing the strength of recurrent links. After the membrane potential reaches the threshold *v*_*th*_, the neuron generates a spike and the voltage resets to *v*_0_. During the absolute refractory period τ_*r*_ after the spike generation, the voltage value remains constant at *v*_0_, i.e., during this interval the neuron is unaffected by external stimulation.

The coupling in (1) is implemented via the double exponential synaptic filter given by the dynamics of variables *r*_*i*_ and *h*_*i*_ for the *i*-th neuron:


(2) $$\begin{array}{l} \begin{array}{c} \frac{dr_{i}}{dt}=-\frac{r_{i}}{\tau_{d}}+h_{i},\\ \frac{dh_{i}}{dt}=-\frac{h_{i}}{\tau_{r}}+\frac{1}{\tau_{d}\tau_{r}}\underset{t_{k}^{\left(i\right)}<t}{\sum }\delta \left(t-t_{k}^{\left(i\right)}\right), \end{array} \end{array}$$

where τ_*r*_ and τ_*d*_ are the synaptic rise and decay time constants, respectively, $t_{k}^{\left(i\right)}$ is the moment of generation of the *k*-th spike by the *i*-th neuron.

The coupling structure of recurrent connections is described by the weight matrix **A** = [*a*_*ij*_] whose elements are drawn from a Gaussian distribution with zero mean and standard deviation *g*(*pN*)^−1/2^ where *p* is a fraction of non-zero elements, *g* is a global coupling strength. The output is given by *M* readout units whose dynamics are determined as follows:


(3) $$\begin{array}{l} {ẑ}_{k}\left(t\right)=\overset{N}{\underset{i=1}{\sum }}w_{ki}r_{i}\left(t\right),\;k=1\ldots ,M \end{array}$$

where *w*_*ki*_ is the weight coefficient between the *i*-th neuron and *k*-th output (resulting in the output matrix **W** = [*w*_*ki*_]), and *r*_*i*_(*t*) is the neural firing rate filtered according to Equation (2).

The FORCE method requires that the output units send feedback links to the spiking neurons whose weights are stored in *N* × *M* matrix **U** composed of concatenated vectors **u**_*k*_ (*k* = 1, …, *M*) and whose elements are drawn randomly and uniformly from a uniform distribution between −*q* and *q*, where *q* is a feedback coupling strength. Therefore, the complete system taking into account the recurrent and feedback links is as follows:


(4) $$\begin{array}{l} \begin{array}{c} \tau_{m}\frac{dv_{i}}{dt}=v_{rest}-v_{i}+I_{bias}+\overset{N}{\underset{j=1}{\sum }}\left(a_{ij}+\overset{M}{\underset{k=1}{\sum }}u_{ik}w_{kj}\right)r_{j},\\ =v_{rest}-v_{i}+I_{bias}+\overset{N}{\underset{j=1}{\sum }}\omega_{ij}r_{j}, \end{array} \end{array}$$

where matrix $\Omega =\text{A}+\text{U}{\text{W}}^{T}=\left[\omega_{ij}\right]$ determines the efficient topology shaped by the fixed recurrent and feedback links and the trained output weights.

The goal of training is to modify output weights *w*_*ij*_ in such a way that the linear readout (3) approximates the target signal: ẑ_*k*_(*t*) ≈ *z*_*k*_(*t*). In this study, we use two-dimensional target signals in the form of closed polar figures which have a clear geometrical interpretation in order to study whether it is possible to relate distinct features of output geometry with the hidden geometry of spike pattern produced by (4). Namely, we illustrate our results by four target curves (5–8) for which equations governing their generation in (*x, y*)-plane are as follows:(a) a circle


(5) $$\begin{array}{l} x_{t}=R\cos\left(2\pi f_{1}t\right),\;y_{t}=R\sin\left(2\pi f_{1}t\right), \end{array}$$

(b) two-petal


(6) $$\begin{array}{l} \begin{array}{c} x_{t}=R\sin\left(4\pi f_{1}t\right)\cos\left(2\pi f_{1}t\right),\\ y_{t}=R\sin\left(4\pi f_{1}t\right)\sin\left(2\pi f_{1}t\right),\\ \phi =2\pi f_{1}t\in \left[0,\pi /2\right]\cup \left[\pi ,3\pi /2\right] \end{array} \end{array}$$

(c) three-petal,


(7) $$\begin{array}{l} \begin{array}{c} x_{t}=R\sin\left(3\pi f_{1}t\right)\cos\left(2\pi f_{1}t\right),\\ y_{t}=R\sin\left(3\pi f_{1}t\right)\sin\left(2\pi f_{1}t\right), \end{array} \end{array}$$

and (d) four-petal


(8) $$\begin{array}{l} \begin{array}{c} x_{t}=R\sin\left(4\pi f_{1}t\right)\cos\left(2\pi f_{1}t\right),\\ y_{t}=R\sin\left(4\pi f_{1}t\right)\sin\left(2\pi f_{1}t\right), \end{array} \end{array}$$

polar roses.

### 4.2 Force training

The output weights in matrix **W** are trained according to the algorithm of first-order reduced and controlled error (FORCE) learning adopted to spiking neural networks, see Sussillo and Abbott (2009) and Nicola and Clopath (2017). The error **e**(*t*) between the teaching signal and the real output is computed after each time interval of Δ*t*:


(9) $$\begin{array}{l} \text{e}\left(t\right)=ẑ\left(t\right)-\text{z}\left(t\right)={\text{W}}^{T}\left(t\right)\text{r}\left(t\right)-\text{z}\left(t\right). \end{array}$$

In addition, a running estimate of the inverse of the correlation matrix of the network rates **P** is computed as follows:


(10) $$\begin{array}{l} \text{P}\left(t\right)=\text{P}\left(t-\Delta t\right)-\frac{\text{P}\left(t-\Delta t\right)\text{r}\left(t\right)\text{r}{\left(t\right)}^{T}\text{P}\left(t-\Delta t\right)}{1+\text{r}{\left(t\right)}^{T}\text{P}\left(t-\Delta t\right)\text{r}\left(t\right)}, \end{array}$$

where matrix **P** is initialized by **I**/α in which **I** is the identity matrix and α is a learning rate parameter. Moreover, after each time period of Δ*t* matrix, **W** is trained according to the following rule based on (9, 10):


(11) $$\begin{array}{l} \text{W}\left(t\right)=\text{W}\left(t-\Delta t\right)-\text{P}\left(t\right)\text{r}\left(t\right)\text{e}{\left(t\right)}^{T}. \end{array}$$

Initially, the elements of output matrix **W** equal to zero, and after each interval, Δ*t* changes with the adaptation rule according to Equation (11). Gradually, the values **w**_*k*_ become close to some stationary states. After that, the learning procedure stops, and we have a multidimensional dynamical system of a complex network with fixed weights. This supervisely trained system is able to autonomously generate the target output closed trajectories. The core structure of the network defined by the adjacency matrix **A** after learning remains the same as before learning. The trained vectors **w**_*k*_ multiplied by the feedback vectors **u**_*k*_ introduce some low-rank perturbation to the coupling topology, and the corresponding network activity dramatically changed. Such structural perturbation leads to a global disturbance in the phase space of the recurrent network.

### 4.3 Victor–Purpura distance for spike trains

Each spike train is considered to be a point in an abstract topological space. A spike train metric is defined according to the special rule, which assigns a non-negative number to pairs of spike trains and expresses how dissimilar they are (Guidolin et al., 2022). We use the variant of the spike time VP distance which is parametrized by the cost quantity *q* in units of the inverse time. To compute the VP distance, the spike trains are compared in terms of allowed elementary steps, which can be applied to one sequence of spike timings to get another one. The following steps and associated costs are as follows: (a) insertion of a spike with the cost of one, (b) deletion of a spike with the cost of one, and (c) shifting a spike by amount of time *t* with the cost of *qt*. If *q* is very small, the metric becomes the simple spike count distance. If *q* is very large, all spike trains are far apart from each other, unless they are nearly identical. For intermediate values of *q*, the distance between two spike trains is small if they have a similar number of spikes, occurring at similar times. The motivation for this construction is that neurons which act like coincidence detectors should care about this metric. The value of *q* corresponds to the temporal precision 1/*q* of the coincidence detector. We calculate the VP distance of the described types using the scripts provided by the authors of this metric: http://www-users.med.cornell.edu/~jdvicto/metricdf.html, http://www-users.med.cornell.edu/~jdvicto/spkdm.html.

Each matrix of VP distances *D*_*ij*_ is transformed via rank-ordering its entries, i.e., we replace the original entries in the above-diagonal part by natural numbers 0, 1, …  in ascending order of their value (Giusti et al., 2015). The below-diagonal part of the rank-ordered matrix is obtained according to the symmetrical transformation of the above-diagonal part. After that, the entries are normalized by *N*(*N* − 1)/2 and reindexed in descending order of the neural firing rates. Finally, we obtain the normalized rank-ordered matrix **M** = [*M*_*ij*_] which contains non-linearly transformed VP distances while having unchanged their relative order.

### 4.4 Persistence barcodes and Betti curves

For the normalized rank-ordered matrices, we perform persistence homology analysis of the following form. The set of 100 most active neurons with their spike trains are considered as vertices in an abstract space, where the distance between the *i*-th and *j*-th neurons are given by entry *M*_*ij*_.

These vertices form zero-dimensional or 0-simplices, and we introduce a filtration parameter ρ which defines the radius of abstract circles centered at vertices. With increasing ρ, two vertices *i* and *j* considered coupled if *M*_*ij*_ ≤ ρ. The edge resulting from such construction is one-dimensional or 1-simplex. With increasing filtration parameter ρ, the number of 0-simplices and 1-simplices changed but may be unchanged or persisted over some intervals. This property is quantified by so-called Betti numbers, which count the number of correspondinc topological invariants at the current filtration scale. For example, the 0-th Betti number β_0_(ρ) gives the number of connected components and the 1-st Betti number β_1_(ρ) counts the number of one-dimensional simplices (edges). How particular simplex (*k*) emerges and disappears is reflected in the persistence barcode which consists of bars $\left[\rho_{b}^{\left(k\right)},\rho_{d}^{\left(k\right)}\right]$, indicating the birth ρ_*b*_ and death ρ_*d*_ values of the filtration parameter for that simplex. Betti curves β_0_(ρ) and β_1_(ρ) summarize this information showing how the number of simplices of corresponding dimensions varies with increasing filtration parameter.

## Data availability statement

The original contributions presented in the study are included in the article/supplementary material, further inquiries can be directed to the corresponding author.

## Author contributions

OM: Conceptualization, Data curation, Formal analysis, Funding acquisition, Investigation, Software, Visualization, Writing – original draft, Writing – review & editing. MP: Funding acquisition, Methodology, Project administration, Supervision, Writing – review & editing. VN: Conceptualization, Investigation, Supervision, Writing – review & editing.
